# Developmental lines of least resistance predict standing genetic covariation but do not constrain plasticity or rapid evolution

**DOI:** 10.1098/rspb.2025.1039

**Published:** 2025-09-03

**Authors:** Patrick Rohner

**Affiliations:** ^1^ Department of Ecology, Behavior, and Evolution, University of California San Diego, La Jolla, CA, USA

**Keywords:** developmental bias, developmental integration, evolvability, **G**-matrix, **D**-matrix, developmental plasticity

## Abstract

Some phenotypic dimensions are more developmentally variable than others. Such developmental variability (or bias) is common and uncontroversial. However, how and at what time scales these biases constrain or facilitate the emergence of standing genetic variation, plastic responses, as well as adaptation remains contentious. To investigate the extent to which developmental variability shapes genetic variation, plasticity and evolution, we first quantify developmental variability in the shape of the dung beetle foreleg—a functional trait critical for the excavation of breeding tunnels. We do so by testing how random developmental perturbations, manifesting themselves in fluctuating asymmetry, shape standing genetic variation within populations. Next, we investigate whether such developmental variability is aligned with thermal plasticity and recently evolved latitudinal variation. We find that, while developmental variability is a strong predictor of standing genetic (co)variance (i.e. the **G**-matrix), latitudinal population differentiation and thermal plasticity were unrelated to developmental variability. This suggests that, while developmental variability may shape standing genetic variation, it does not seem to constrain the evolution of putatively adaptive population differentiation and plastic responses. At least in this system, developmental biases do not seem to constrain morphological differentiation on ecological time scales.

## 1. Introduction

Perturbations of developmental systems—whether they are caused by novel mutations or environmental stress—often affect some traits more than others. Such developmental variability has long been recognized as an intrinsic feature of developmental systems [1–6]. However, although ubiquitous, the role of developmental variability in evolution remains contentious and poorly understood [4,7,8]. Because developmental architectures have the potential to channel random mutational or environmental perturbations into *non*-random phenotypic effects, they shape how novel genetic or environmental variation manifests on the phenotypic level, thereby affecting which phenotypes become visible to selection. Therefore, understanding the role of developmental variability in the accumulation of genetic variation and the evolution of environmental plasticity is important for our understanding of fundamental evolutionary processes [9–11].

An increasing number of studies suggests that developmental variability (or bias) plays an underappreciated role in evolution [1,4,7,12,13]. For instance, studies on fly wings found that those phenotypic dimensions that are most developmentally variable also harbour most standing genetic variation and covariation (i.e. the **G**-matrix) [14,15]. Genetic (co)variation is a key factor in evolution because it determines the capacity of a population to respond to selection on short time scales [16–19]. If developmental variability generally predicts the structure of **G**-matrices, it might thus be a major determinant (or predictor) of the **G**-matrix and its stability over time and space [20], and may, in turn heavily impact the speed and direction of evolutionary responses to selection [17,21–24]. If so, this would emphasize the role of developmental—rather than ‘genetic’—interactions in evolution and place increased emphasis on the evolution and stability of developmental architectures. This idea has recently gained traction as several studies suggest an alignment between developmental variability and macroevolution on time scales up to tens of million years [15,25–27]. The emerging pattern suggests that developmental covariation might be a useful predictor of micro- as well as macroevolutionary dynamics. However, the evolutionary forces behind this congruence remain contentious [24,27], and whether these patterns are common or found only in a few select systems remains unclear.

If developmental architectures affect how genetic perturbations manifest themselves on the phenotypic level, the same may also apply to environmental perturbations [28–30]. Indeed, plasticity has been found to be aligned with developmental covariation in a range of systems, including dung beetle horns [25], macaque skulls [31] and fruit fly wings [32]. These observations are consistent with the hypothesis that developmental architectures shape organismal responses to genetic as well as environmental responses, which may explain why plastic responses to novel environments often recapitulate mutational effects (resulting in ‘phenocopies’ [10,28,33]) and are often aligned with the main dimensions of **G** (i.e. its leading eigenvector, commonly referred to as $g_{max})$ [34,35], although this alignment is highly context-dependent [34,36]. The architecture of the developmental system may thus simultaneously bias plasticity and genetic variation, leading to an alignment between the **G**-matrix and plastic responses to novel environments. If sufficiently strong, this may also drive the alignment between genetic differentiation and plasticity in multivariate characters. This offers an alternative explanation for these alignments which are often interpreted as solely driven by natural selection [37,38].

One way of quantifying developmental variability is to investigate patterns of fluctuating asymmetry [8,25,27]. Because the left and right versions of the same trait in most bilaterians share not only an identical genome but also the same environment, left-right asymmetry (fluctuating asymmetry) can be attributed to stochastic developmental noise. Fluctuating asymmetry thus provides insights into developmental integration and developmental variability [25,26,32,39]. Using this approach not only allows identifying the phenotypic dimensions that are most strongly affected by these random perturbations but also to assess the covariation among traits induced by developmental perturbations (i.e. the developmental covariance matrix, **D**). Similar to the **G**-matrix, which describes how genetic variation shapes trait variation and covariation, the **D**-matrix describes how developmental perturbations affect trait (co)variation. Here, we use this approach to quantify developmental variability in the shape of beetle legs and test whether it is related to genetic covariation, local adaptation, as well as developmental plasticity.

The bull-headed dung beetle *Onthophagus taurus* is native to the Mediterranean region but was accidentally introduced to the southeastern United States in the 1970s [40] and has since drastically increased its range. While the species was first recorded in northern Florida, it can now be found across the Eastern United States [41,42]. This rapid range expansion coincided with the evolution of life-history plasticity and functional morphology [43,44]. For instance, females develop particularly broad shovel-like fore tibiae used to excavate subterranean breeding tunnels [45,46]. The shape of the tibia shows plastic as well as genetic latitudinal variation. Specifically, relative tibia width increases when females are reared at low temperatures. Similar responses are found along a latitudinal gradient with higher latitude populations developing broader tibiae [43]. Although the precise function in the field remains to be investigated, the female tibia is an ideal functional trait to investigate how developmental variability shapes plasticity and rapidly evolving population differentiation.

To investigate the role of developmental variability in the structuring of standing genetic covariation (the **G**-matrix), plasticity and microevolution, we quantify fluctuating asymmetry in tibia shape and ask whether developmental covariation predicts the **G**-matrix, latitudinal population differentiation, as well as thermal plasticity. If developmental architectures bias both genetic and environmental effects, we expect **D** to be aligned with **G**, latitudinal population differentiation, as well as plastic responses. While our data suggest that developmental variability indeed shapes the standing genetic covariation visible to selection, it does not seem to constrain local adaptation or thermal plasticity.

## 2. Methods

### (a) Laboratory rearing and data acquisition

**Figure 1.**
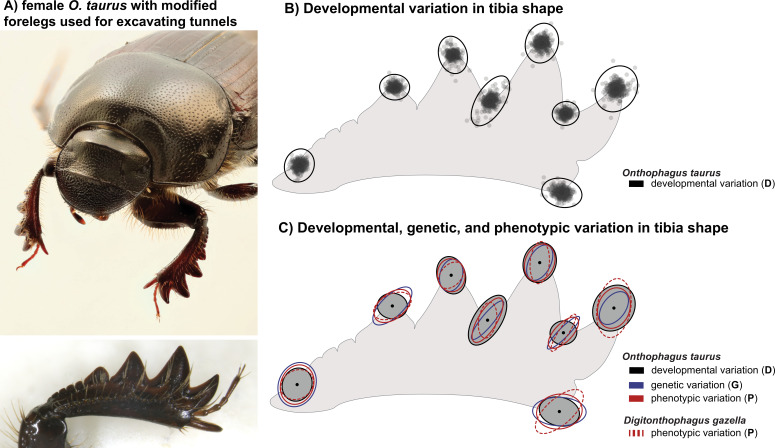
Morphological variation in the fore tibia of female *Onthophagus taurus*. (A) An adult female with exaggerated, shovel-like fore tibiae, used for excavating underground breeding tunnels. (B) Visualization of fluctuating asymmetry in landmark positions. Each point represents the average deviation in landmark position between the left and right sides of an individual magnified 10-fold (for visualization). Standard deviation ellipses are drawn based on the variances and covariances as estimated in the developmental covariance matrix **D**. Ellipses reflect the variance and covariance among the *x* and *y* components of each landmark and are magnified 19.6 fold. Panel C shows the alignment of developmental, genetic and phenotypic variation in fore tibia shape. For comparison, all covariance matrices were scaled to the same total variance as **D** and magnified 10-fold. The phenotypic covariance matrix from a second dung beetle species (*Digitonthophagus gazella*) is shown with hatched ellipses.

To investigate how developmental variability relates to genetic and environmental variation, we revisited a common garden study conducted by Rohner & Moczek [43]. In brief, this study sampled *O. taurus* (figure 1A) beetles from one location in the native Mediterranean range (Italy) as well as four populations in the invasive range in the United States. Wild-caught females were brought into the laboratory where they were then allowed to reproduce in plastic containers (27 cm × 8 cm × 8 cm) filled with soil and defrosted cow dung. Reproductively active females excavate tunnels in the soil and construct so-called ‘brood balls’ underground. These brood balls were extracted from the soil using a sieve after 5 days. Because larval growth and adult morphology strongly depend on the size and quality of the brood ball, eggs were removed from their natural brood ball and placed in standardized artificial brood balls (consisting of cow dung placed in 12-well plates). The brood of each female was then split evenly and kept in incubators at constant 19℃ or 27℃. These temperatures were chosen as they approximate average soil temperatures during the breeding season at the northern and southern range edges in the exotic range (Florida versus Michigan). After adult emergence, beetles were sacrificed and stored in 70% ethanol. This common garden rearing generated 679 individuals, including 321 females. We focused on females because this is the sex that primarily excavates tunnels in this species and correspondingly evolved an exaggerated tibia morphology [45].

To increase the sample size for the estimation of developmental variability, we added an additional 113 laboratory-reared F1+ females that were originally collected in North Carolina (the same location as in the common garden experiment). This led to a sample size of *n* = 434 for *O. taurus*. In addition to this focal species, we also added an additional 83 female *Digitonthophagus gazella*. This species is morphologically and ecologically similar but shared its most recent common ancestor with the *Onthophagus* lineage about 40 million years ago (40 Ma) [47]. This species was added to provide an additional point of comparison for the statistical comparison of covariance matrices (see below).

We removed the left and right foretibiae of all beetles and photographed them using a Pixelink camera (M20C-CYL) mounted on a Leica M205 stereoscope. For each photograph, the foretibia was placed on a piece of modelling clay and positioned in such a way under the stereoscope that all features that were used for landmarking were in focus. Each tibia was photographed twice. The tibia was removed from the clay and positioned anew between pictures to acquire two independent measurements of the same sample. We then placed eight two-dimensional landmarks (figure 1B) on the pictures using TpsDig2 [48]. Because each landmark has *x* and *y* components, these 8 landmarks are represented by 16 different sets of coordinates. This led to a final dataset for *O. taurus* of 1736 measurements of 16 variables (434 individuals in total × 2 sides (left/right) × 2 independent measurements). All landmark coordinates were then aligned simultaneously using Procrustes superimposition in the R-package *geomorph* [49]. This method is used in geometric morphometrics to align shapes such that they can be meaningfully compared without the confounding effects of scaling, position and rotation [50]. All the following procedures are based on the Procrustes shape coordinates. Centroid size was extracted as a measure of structural size. A Procrustes analysis of variance (ANOVA) (implemented in the *geomorph* function *bilat.symmetry()*) was used to quantify the proportion of the total variance explained by individual differences (individual main effect), directional asymmetry (side main effect), fluctuating asymmetry (individual-by-side interaction), as well as measurement error (electronic supplementary material, table S1).

Note that, while the original study Rohner & Moczek [43] also focused on tibia shape, the analysis here is based on a completely independent set of pictures and a slightly modified set of landmarks to enable comparisons across species (see below).

### (b) Estimating the developmental, genetic and phenotypic variance matrices

We estimated developmental variance matrix **D** for tibia shape using mixed models in ASReml-R [51,52]. We fitted all tibia shape variables simultaneously using the individual, side (directional asymmetry) and log centroid size (accounting for allometry) as fixed effects. The **D**-matrix was estimated based on the interaction term between individual and side. The **D** matrix thus captures the variance and covariance among landmarks that are caused by developmental variability within an individual. Note that, for this analysis, we pooled individuals across populations, temperature treatments and rearing regimes (common garden versus laboratory). Any genetic or environmental effect on tibia shape is captured by adding individual identity as fixed effect (thereby taking into account differences among treatments, populations, etc.).

To calculate the phenotypic variance matrix, **P**, we used similar models that included log centroid size, temperature, side and population as fixed effects. The covariance matrix was calculated based on the random effect of individual identity. The **P-**matrix thus captures patterns of variance and covariance in tibia shape among individuals, while accounting for the effects of rearing temperature, directional asymmetry, allometry and population differentiation (this type of covariation is commonly referred to as ’static’ covariance [53]).

As an estimate of the broad-sense genetic variance-covariance matrix **G**, we first calculated the average tibia shape per individual (based on the measurements taken on the left and right sides) and then fitted a model using log centroid size, population and rearing temperature as fixed effects while estimating the covariance matrix based on the random effect of (full sibling) family identity. This matrix contains the variances and covariances among shape variables that are driven by genetic effects (including both additive and non-additive effects). Family was treated as a categorical variable. We did not have the statistical power to assess standing genetic variation within each of the studied populations. Consequently, this matrix represents the pooled within-population genetic covariation.

The error variances in all the models used to estimate **D**, **P** and **G** were estimated for each shape variable separately in order to accommodate landmark coordinate specific measurement error.

### (c) Estimating the dimensionality of covariance matrices

As is common for morphometric data, variance–covariance matrices are highly redundant and rank-deficient [54]. To assess the dimensionality of **D** and **G**, we fitted reduced-rank factor analytic mixed models in ASReml-R [15,55]. We began by fitting a covariance model with only one dimension and then increased the dimensionality until Akaike’s Information Criterion (AIC) indicated that further increasing the number of dimensions did not lead to a significant increase in model fit or models stopped converging. We then used the function *fa.asreml()* from the R package ASExtra4 [52] to reconstruct the (co)variance structure from factor analytical models. This step was necessary to facilitate interpretation and visualization, which is otherwise challenging for factor analytical models.

### (d) Comparison of variance matrices

To test whether those phenotypic dimensions with highest developmental variation are also those that show most standing genetic variance, we used a modified version of Krzanowski’s common subspace analysis following the method described in [56] (see also [14,15,57]). This analysis compares the logarithmized variances of both matrices along the same set of orthogonal vectors $K_{D}$. The variances along each dimension were calculated by taking the diagonal entries of the matrix $K_{D}^{T}DK_{D}$ and $K_{D}^{T}GK_{D}$ (for the **D** and **G**-matrices, respectively). If **D** and **G** are closely related, they are expected to show similar levels of variance in any direction, irrespective of what set of orthogonal vectors is being used for comparison. We therefore calculated Pearson’s correlation coefficients (*r*) between these logarithmized developmental and genetic variances along each axis. The strength of this correlation then relates to the degree to which developmental variability (**D**) predicts standing genetic variation (**G**).

Because the choice of the eigenvectors along which variances are compared can affect the observed alignment [56,57], we compare the variances along the eigenvectors of an independently generated third matrix—the phenotypic variance–covariance matrix, **P**. As an additional independent comparison, we also estimated the **P** matrix in the gazelle dung beetle *D. gazella*. This species diverged from *O. taurus* approximately 40 Ma [47] but is morphologically similar. As none of our matrices were full-rank, we restricted the calculation of correlations and slopes to the first six dimensions, representing the maximum number of ranks shared across all matrices compared (electronic supplementary material, table S2).

To take into account error of the estimation of matrix estimation, we used the REML-MVN approach [58] to calculate approximate 95% confidence limits around correlations and slopes. We performed the MVN resampling on the ‘G-scale’ using the *mvtnorm* package for R (see also [59,60]). With this approach, we resampled 10 000 **G**, **P** and **D**-matrices and subjected them to the common subspace analysis. We then calculated bias‐corrected and accelerated bootstrap (BCa) confidence intervals around the correlation and slope based on the comparison of these resampled matrices.

In addition to the common subspace analysis, we also computed the vector correlation (or cosine similarity) between the first eigenvectors of **G** and **D** ($g_{max}$ and $d_{max}$, respectively) as


$$r=\cos\theta =\;\frac{\left|d_{max}\;\cdot \;g_{max}\right|}{\left\|d_{max}\right\|\;\times \;\left\|g_{max}\right\|}$$

where the numerator denotes the dot product of the two eigenvectors and the denominator represents the product of their norms. Confidence limits were generated by computing the vector correlation for all 10 000 matrices generated by MVN resampling (see above). If the primary axes of developmental and genetic variation are aligned, this correlation is expected to be high.

As an additional metric of matrix similarity, we also used ‘random skewers’ [61] as implemented in the R package *EvolQG* [62]. In brief, this method takes advantage of classic quantitative genetic theory [17] to quantify the similarity in matrix structure by applying a large number of random selection vectors to each matrix, computing the response vectors for each matrix, and then quantifying the average correlation between corresponding responses. Matrices that produce similar responses across random vectors are considered structurally similar. In our analysis, we used 10 000 vectors of unit length drawn from a uniform distribution to make these comparisons.

### (e) Quantifying the alignment between **D** and latitudinal population differentiation and thermal plasticity

The latitudinal vector was estimated by a regression of average tibia shape of each family (across temperatures) as a function of latitude as


$$Y\;∼\;\beta_{0}+\beta_{C}\times Centroid+\beta_{L}\times Latitude+\;\varepsilon .$$

where Y is a matrix of Procrustes shape variables averaged by family across temperature treatments, $\beta_{0}$ is the intercept, $\beta_{C}$ and $\beta_{L}$ are the vectors of regression coefficients for centroid size and latitude, respectively. ε is a vector of error terms.

The effect of temperature on tibia shape was estimated from the following model


$$Y\;∼\;\beta_{0}+\beta_{C}\times Centroid+\beta_{T}\times Temperature+\beta_{P}\times Population+\;\varepsilon ,$$

where Y is the matrix of shape variables averaged by individual. $\beta_{C}$, $\beta_{T}$ and $\beta_{P}$ are the vectors for the effects of centroid size, temperature, and population.

To test whether plastic and evolutionary responses are more closely related to **D** than expected by chance, we first quantified the alignment of these shape change vectors with **D** as


$$e_{\beta }=\;\frac{\beta^{T}D\beta }{{\left|\beta \right|}^{2}}$$

where β is the shape deformation vector of interest (i.e. the vector associated with the latitudinal cline ($\beta_{L}$) or thermal plasticity ($\beta_{T}$)), **D** is the **D**-matrix scaled by its trace and *T* denotes transposition. This measure ($e_{\beta }$) gives the amount of variation in **D** captured by the shape vectors. If the shape changes associated with the latitudinal cline or thermal plasticity occur primarily along the main axes of **D**, $e_{\beta }$ is expected to be large and similar in magnitude to the first eigenvalue of **D**. To assess whether the observed value of $e_{\beta }$ is larger than expected by chance, we generated a null distribution by resampling the shape vectors and comparing the observed $e_{\beta }$ to the distribution of the resampled values.

As an additional metric of alignment, we also calculated the angle between the shape change vector and $d_{max}$ and $g_{max}$. The significance of this angle was assessed by resampling the observed vectors 10 000 times and comparing the observed angle to the distribution of these randomized angles.

## 3. Results

### (a) Developmental variability in tibia shape

We sought to characterize developmental variability in tibia shape by investigating fluctuating asymmetric components of shape variation. Although the female foreleg resembles a digging appendage playing a key role during reproduction [45,46] and is thus expected to be under strong selection, we found significant fluctuating asymmetry in tibia shape accounting for 11 percent of the total phenotypic variation (Procrustes ANOVA, individual × side interaction: *F*_433,868_ = 4.71, *Z* = 18.21 and *p* = 0.001). This asymmetry was mostly related to the relative height of the tibial teeth (figure 1). AIC-based model comparisons of different factor analytical models showed that fitting a seven-dimensional **D**-matrix had the lowest AIC, indicating that our **D**-matrix is rank-deficient, but that there is significant developmental variability along seven orthogonal phenotypic dimensions.

### (b) Relationship between developmental and genetic covariation

**Figure 2.**
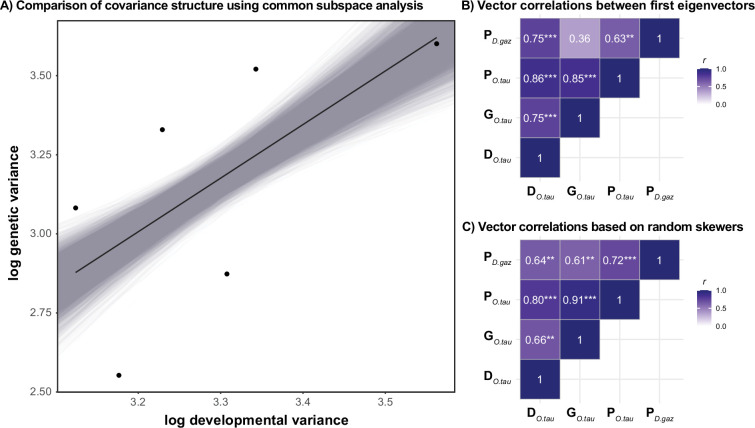
Comparison of matrix structure. (A) The amount of developmental variance along a set of six vectors are correlated with the amount of genetic variance along the same set of vectors. Black points and regression line represent observed values. Grey lines indicate the distribution of linear regressions of all resampled data (generated using REML-MVN resampling). We used the first six eigenvectors of the statistically independent phenotypic covariance matrices in *Onthophagus taurus* as comparison. Using the **P** matrix in the gazelle dung beetle, which diverged from *O. taurus* around 40 Ma rendered similar results. Panels (B) and (C) show the pairwise correlation coefficients between the dominant eigenvectors among the **D**, **G** and **P** matrices, as well as the results of a random skewers analysis (**p < 0.01; ***p < 0.001).

In addition to asymmetry, we also found heritable variation in tibia shape. Including full-sib family as random effect greatly increased model fit (*χ*^2^_97_ = 784.9, *p* < 0.001). A model fitting a six-dimensional **G**-matrix had the lowest AIC, suggesting that at least six dimensions have heritable genetic variation (models with higher dimensionality did not converge). When using the first six eigenvectors of the **P** matrix as a reference for comparison, we find that the amount of variance along these vectors captured by **D** predicts how much variance is captured in **G** (*r* = 0.87 [0.75, 0.94], *b* = 1.90 [1.44, 2.30]; figure 2A). Phenotypic dimensions with more developmental variation thus also harbour more genetic variation. Similar, yet weaker, alignments are found when using the **P**-matrix estimated in a species that diverged from the focal species *ca* 40 Ma (*D. gazella*: *r* = 0.62 [0.48, 0.85], *b* = 0.39 [0.30, 0.66], number of dimensions = 6).

Complementary approaches led to similar conclusions. For instance, the main axis of genetic variation ($g_{max})$ was associated with the phenotypic dimension that had the highest developmental variability ($d_{max}$) (θ = 41.5° [33.3, 81.2] 95% CI, r = 0.75 [0.15, 0.84] ; figure 2B). There was also a significant similarity in matrix structure based on the random skewers analysis (*r* = 0.66, *p* = 0.002; figure 2C). Taken together, these different approached suggest that standing genetic covariance patterns are related to developmental variability.

### (c) Alignment between developmental variability, thermal plasticity, and latitudinal cline

**Figure 3.**
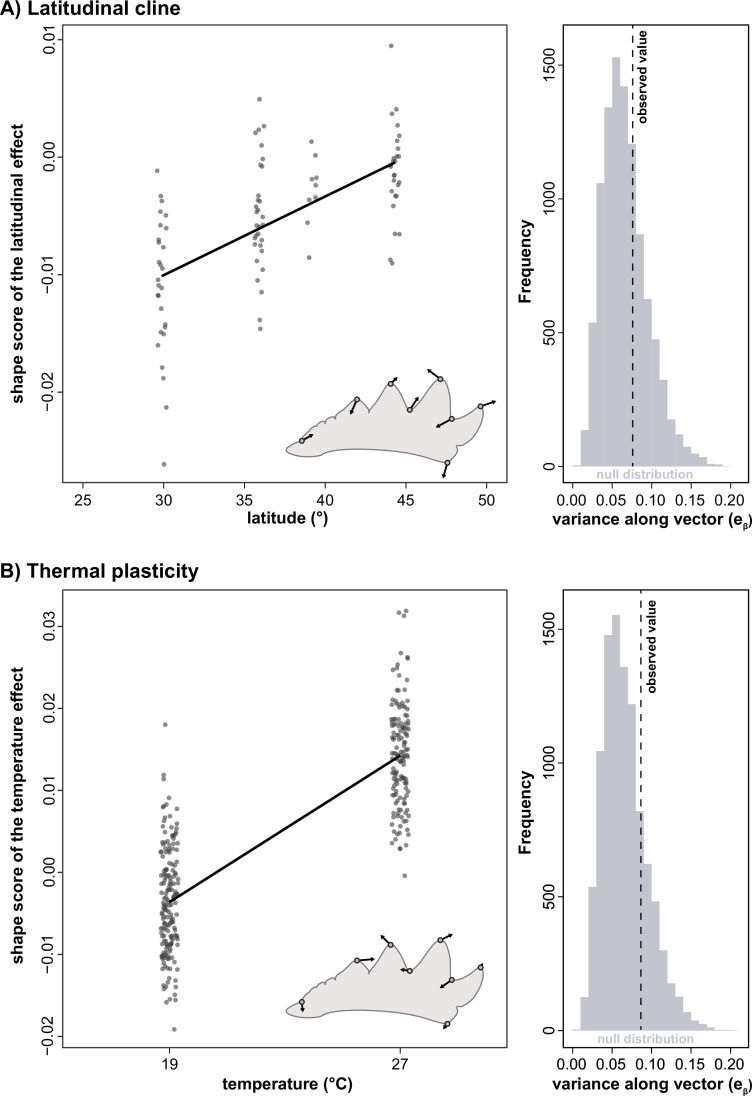
Lack of alignment between latitudinal clines or thermal plasticity and the developmental covariance matrix. (A) Tibia shape exhibits latitudinal clines across the invaded range [43]. Shape scores were calculated by projecting individual shapes onto a vector associated with latitude following [63]. Insets illustrate changes in landmark positions associated with increasing latitude. The right-hand plot shows the proportion of variance in the developmental covariance matrix (**D**) explained by the latitude-associated shape change vector. The grey histogram represents the distribution of variance explained by randomized shape vectors. (B) Effects of rearing temperature on tibia shape [43], and the variance in **D** captured by the observed shape change due to thermal plasticity. Again, the grey histogram reflects a randomized distribution for comparison. Overall, shape changes associated with both latitude and temperature are not more aligned with the developmental covariance matrix than expected by chance.

Given that we found an alignment between **D** and **G**, we next investigated whether developmental variability (**D**) also predicts plastic variation and putatively adaptive microevolutionary diversification. We found significant effects of rearing temperature on tibia shape, as well as a genetic latitudinal cline (as shown previously [42]). However, the shape vector associated with thermal plasticity was not aligned with $d_{max}$ (θ = 80.8°, *r* = 0.16, *p* = 0.466) and only explained 8.7% of the total variation in **D** (*p* = 0.211, figure 3). Similarly, the latitudinal shape change vector was not aligned with $d_{max}$ (θ = 80.3°, *r* = 0.17, *p* = 0.459) and did not explain more variation of **D** than expected (proportion of trace = 0.076, *p* = 0.321, figure 3). Neither shape vector was aligned with the **G**-matrix. In contrast to the alignment between **D** and **G**, we therefore found little evidence for an alignment between **D** with plasticity or the latitudinal cline.

## 4. Discussion

The architecture of developmental systems may bias the effects of genetic and environmental perturbations, thereby potentially steering evolution in certain directions. The degree to which developmental variability constrains or facilitates adaptation and how variability itself evolves is thus central for our understanding of fundamental evolutionary processes [2,4,64,65]. Studying morphological variation in the fore tibia of female dung beetles, we find that developmental variability predicts standing genetic covariation within populations (i.e. the [broad-sense] **G**-matrix). Through its influence on genetic covariation, developmental variability may thus affect the speed and direction of evolutionary divergence [20,21,24]. However, developmental covariation was not aligned with thermal plasticity or recently evolved latitudinal population differentiation. Developmental variability therefore does not necessarily constrain the evolution of developmental plasticity or the rapid evolution of putatively adaptive clinal variation. This contrasts with several previous studies suggesting that developmental variability aligns with putatively adaptive phenotypic variation [13,15,27]. Developmental variability thus acts as a facilitator of genetic variation but does not *per se* limit local adaptation.

### (a) Developmental variability and evolvability

The **G**-matrix is a central quantitative genetic parameter predicting evolutionary responses of multiple polygenic traits to selection [17,18,23,66]. While genetic variances (the diagonal entries of **G**) are driven by mutation, selection, and gene flow, the genetic co-variances (i.e., the off-diagonal elements of **G**) can be driven by pleiotropy and linkage. **D**, on the other hand, is driven exclusively by developmental interactions (assuming that the influence of somatic mutation is negligible). The similarities between **G** and **D** suggest that pleiotropic interactions are the dominant force shaping the orientation of the **G**-matrix. This would argue for a major role of development in shaping genetic covariation and, consequently, trait evolvability (i.e. **D** directly influences **G**, which in turn affects responses to selection). Alternatively, the resemblance between **D** and **G** could be a result of correlational selection shaping both independently to align with the adaptive landscape (e.g. [64]). Several recent studies argued that the alignments between developmental (**D**), mutational (**M**), genetic (**G**) or even macroevolutionary (**R**) covariation may be due to such multivariate selection shaping optimal trait combinations across levels of biological organization [14,15,67,68]. Given their role in the excavation of breeding tunnels [45] and coevolution between tibia shape and tunnelling behavior [46], selection on tibia morphology is likely to be strong and persistent. It is thus plausible that selection simultaneously shapes both **D** and **G**. The alignment between **P** matrices in *O. taurus* and *D. gazella* (figure 2) suggests that patterns of covariation are stable across tens of millions of years, possibly due to shared selection pressures given their overall similar ecology relating to tunneling behaviour. Such pervasive patterns of ecological selection might drive the evolution of **D** and **G** matrices. However, we currently lack estimates of multivariate selection on tibia shape and the extent to which developmental variability responds to selection is unknown. Whether **D** directly shapes **G** or whether both are simultaneously driven by selection thus remains unclear and will require further investigation.

### (b) Developmental variability does not constrain plasticity or latitudinal differentiation

Similar to the way developmental interactions may shape the phenotypic effects of novel mutations, it may also affect the phenotypic consequences of environmental variation [30,33]. We thus expected thermal plasticity to align with developmental variability, as has been found in other traits and species [25,32,34]. Contrary to these expectations, thermal plasticity was not aligned with the **D**-matrix. The experimental treatments applied cover a large temperature range (19℃ versus 27℃), but this is within the range that is experienced by populations in the field [69]. It is thus likely that thermal plasticity in tibia morphology across this temperature range is the result of selection. Tunneling behaviour is a synapomorphy that dates back to the origin of the dung beetle clade around 50−130 Ma [70,71] and similarly exaggerated forelegs are found in a large number of species (e.g. [72]). Selection on plasticity in relation to temperature might thus be fairly old and, given the time scales involved, **D** may not be expected to pose any hard constraints on the evolution of plasticity.

A more intriguing finding was the lack of alignment between the recently evolved latitudinal variation in tibia shape and developmental variability. Given the establishment of clinal variation over a short timeframe (80–100 generations [69]), developmental (and genetic) variability would be expected to constrain population differentiation. Yet, the latitudinal cline was unrelated to the direction of the **D**-matrix. While developmental biases may influence genetic covariation, there thus remains sufficient genetic variability to facilitate adaptive changes. One scenario in which developmental variability may not pose a major constraint on adaptation may be if evolutionary changes are driven by genetic accommodation (i.e. an evolutionary modification of an already existing plastic response [10,73–75]). In these cases, evolutionary change is expected to modify already existing ancestral developmental interactions. In cases where plasticity precedes (and leads) adaptation, constraints imposed by developmental variability may be more easily overcome.

The alignments between **D** and **G**, as well as the lack of association with thermal plasticity, suggests that the influence of developmental bias on evolution in beetle forelegs might be overall weaker than in the previously studied fly wings [14,15]. This is unexpected because both traits play important functional roles in locomotion and reproduction. One potential cause of this discrepancy is the stark difference in how fly and beetle appendages develop during metamorphosis. Beetle tibiae develop during the pupal stage from polymorphic larval cells [76,77]. That is, the pupal and adult legs develop during metamorphosis from cells that previously constituted the larval leg. In contrast, fly appendages follow a highly derived mode of metamorphic development. Fly wings and legs develop from imaginal discs which consist of specialized epithelial tissue set side during embryogenesis [78,79]. Imaginal discs grow during the larval stage but they only start to differentiate until the pupal stage. In contrast to beetle legs, the tissues that generate the adult fly wings thus do not function in locomotion in the larval stage, and they undergo much more extreme morphological changes during a much shorter period of time [79]. The rapid formation of fly wings may thus require a more robust developmental programme that leads to much stronger developmental biases in fly wings compared with dung beetle legs, leading to stronger alignments between **D** and plasticity.

## 5. Conclusions

Developmental variability is ubiquitous but the part it plays in evolution remains contentious. We find that those phenotypic dimensions that have a high degree of developmental variance also harbor more genetic variance. This suggests that developmental variability has the potential to influence the speed and direction of microevolution, suggesting the existence of ‘*developmental*’—rather than ‘*genetic*’—lines of least resistance [21]. However, this alignment does not seem to enforce hard constraints on the rapid evolution of putatively adaptive clinal variation and thermal plasticity. The degree to which developmental variability can predict phenotypic variation may thus depend on the strength of selection and the time scales considered.

## Data Availability

Raw data are available on Dryad [80]. Supplementary material is available online [81].
